# Blood Cell-Based Drug Delivery Systems: From Biological and Mechanical Design to Clinical Applications

**DOI:** 10.3390/bioengineering13030340

**Published:** 2026-03-14

**Authors:** Gang Xu, Xuejin Li

**Affiliations:** 1 Department of Engineering Mechanics, Zhejiang University, Hangzhou 310027, China; xugangtcm@163.com; 2 Department of Endocrinology and Metabolism, The First Hospital of Ningbo University, Ningbo 315010, China

**Keywords:** cell-based drug delivery, blood cells, cellular hitchhiking, cell carrier, nanoparticle

## Abstract

Conventional drug delivery systems often suffer from problems such as limited targeting specificity, short half-lives, poor biocompatibility, and systemic toxicity, which significantly limit their therapeutic efficacy against major diseases like cancer. Blood cells, as native components of the human circulatory system, offer distinct advantages including low immunogenicity, long circulation times, remarkable mechanical flexibility, and innate ability to home to disease sites. These attributes make blood cells a promising platform for next-generation targeted drug carriers. In this review, we examine the biological and mechanical properties of red blood cells, white blood cells, platelets, and cell-derived membrane vesicles. We highlight recent advances in how these cells are engineered and loaded with drugs, and their application in tumor-targeted therapy, while also considering their potential in other diseases. We also discuss current technical challenges and outline future directions for clinical translation, offering a practical perspective on advancing blood cell-based delivery technologies.

## 1. Introduction

The main goal of drug delivery systems (DDS) is to deliver therapeutics precisely to target sites, control their release, maximize efficacy, and minimize harm to healthy tissues [1,2,3]. This precision is especially critical in cancer therapy, as clinical studies indicate that only a small fraction of intravenously administered anticancer drugs reach tumor sites, with the majority being cleared by the liver and spleen during circulation. This leads to inefficient drug use and severe side effects such as myelosuppression and organ damage [4,5]. In recent years, the convergence of nanomedicine, cell engineering, and biomechanics has opened new ways for bio-inspired drug delivery [6,7,8]. Among these, blood cell-based DDSs are particularly interesting [9,10,11]. They leverage inherent biological advantages that synthetic carriers often struggle to replicate.

**Figure 1:**
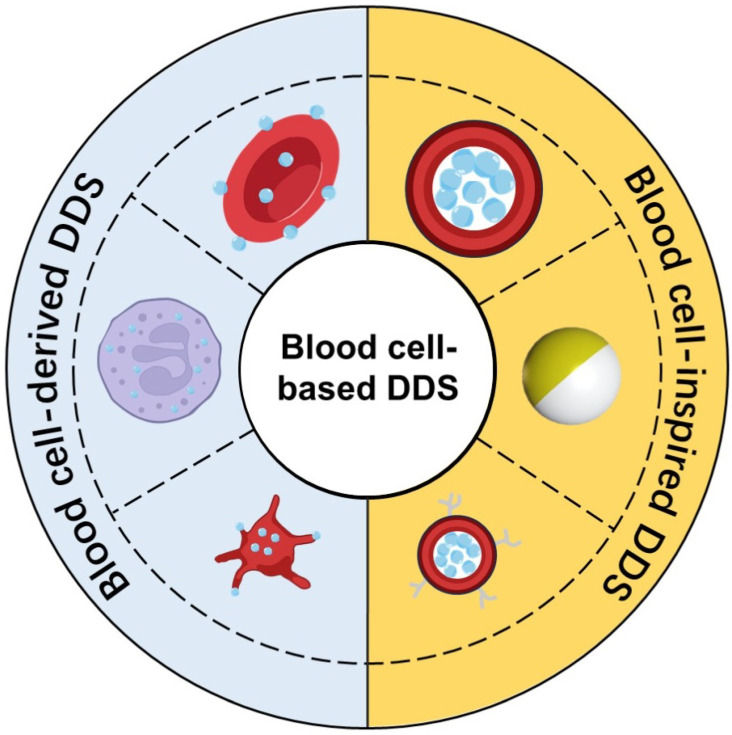
Schematic representation of blood cell-based drug delivery systems. Left panel (cyan background): Natural blood cells (RBCs, WBCs, and platelets) used directly as drug carriers. Right panel (yellow background): Engineered biomimetic carriers designed to mimic structural and functional attributes of blood cells.

Consider the main cellular components of blood: red blood cells (RBCs), white blood cells (WBCs), and platelets. They exist in different forms and serve different functions, but they share some useful features for drug delivery (Figure 1). RBCs, for instance, are the most abundant cell type. They lack a nucleus and can circulate for up to 120 days. They are also highly deformable, allowing them to squeeze through capillaries much narrower than themselves [12,13,14]. WBCs, especially macrophages and neutrophils, have a natural tendency to migrate toward inflammatory signals, which can originate from tumors, enabling them to actively enter tumor sites [15,16]. Platelets are adept at sensing vascular injury. They accumulate efficiently in the tumor microenvironment and can even cross tight barriers like the blood–brain barrier (BBB) [17,18]. Moreover, using a patient’s own blood cells as carriers generally avoids immune rejection. This offers better biocompatibility and safety profiles compared to many synthetic nanocarriers [19].

As summarized in Table 1, by leveraging inherent advantages such as superior biocompatibility and extended circulation, blood cell-based DDSs have emerged as a highly promising research direction for next-generation targeted delivery [7]. To provide a comprehensive perspective, this review will first detail the unique biological and mechanical properties of blood cells and their membrane-derived vesicles. Subsequently, we will examine the key engineering and drug-loading strategies that make these natural carriers useful for therapeutic applications. Their promising applications in oncology, where their targeting and barrier-penetrating abilities are particularly valuable, will then be highlighted. Finally, we will critically discuss the prevailing technical hurdles and outline potential solutions to advance these systems toward clinical translation.

## 2. Biological and Mechanical Design Principles

As functional units of the circulatory system, blood cells possess intrinsic biological and mechanical properties that support their delivery potential. Differences in their structure and function influence factors such as targeting specificity and drug-loading capacity, while their unique mechanical behavior helps them navigate through blood vessels and into diseased tissues.

### 2.1. RBCs: Long-Circulating Carriers

RBCs are the most abundant cell type in blood, accounting for about 40 to 50% of blood volume. Their structure is well-suited for delivery: with no nucleus or organelles, their interior is mostly hemoglobin, leaving room for loading small molecules, proteins, or even gene vectors [20,21,22]. Their membrane displays ‘don’t eat me’ signals such as CD47. This helps them evade immune clearance and contributes to their long circulation time of up to 120 days, much longer than most synthetic carriers [23,24].

From a mechanical standpoint, RBCs are both durable and flexible. With a diameter of 7–8 μm, they routinely deform to pass through capillaries as narrow as 3 μm, due to an elastic membrane and viscous cytoplasm [12]. This reversible deformation protects them from shear-induced rupture and prevents premature drug leakage. RBCs also have a natural end-of-life homing mechanism: aging cells are cleared by macrophages in the spleen and liver. This process can actually be exploited for spleen-targeted delivery, which could be useful for treating immune disorders or splenic metastases [25,26].

### 2.2. WBCs: Actively Homing Vehicles

WBCs are the body’s mobile immune units, and include various cell types such as macrophages, neutrophils, and lymphocytes [27]. For drug delivery purposes, macrophages and neutrophils stand out because of their natural ability to actively seek out specific targets. Growing tumors release a range of signals (inflammatory factors like TNF-α and IL-6, chemokines like CXCL12, and general damage signals) that recruit these cells from the bloodstream [15,16].

As professional phagocytes, macrophages can engulf nanodrugs or liposomes, acting as Trojan horse carriers to transport them across tumor vascular barriers and through dense stroma, reaching hypoxic regions that are often inaccessible to conventional carriers [28,29]. Evidence supports the potential of macrophage carriers for targeting deep tumors [30,31]. Lymphocytes, especially T cells, can be engineered to perform dual roles: target tumors via receptors like chimeric antigen receptors (CARs) and secrete drugs or cytokines locally [32].

Their mechanical properties support this function. Through surface integrins, they adhere to vessel walls and migrate through endothelial gaps, a process reliant on membrane fluidity and cytoskeleton dynamics [33,34,35]. This allows them to infiltrate leaky tumor vasculature and achieve deep tissue delivery.

### 2.3. Platelets: Nature-Derived Carriers Targeting the Tumor Microenvironment

Platelets are small (2–4 μm), anucleate cell fragments derived from megakaryocytes [36]. Their primary physiological role is to maintain hemostasis by rapidly responding to vascular injury. This response is initiated when platelet surface receptors, including glycoprotein Ib-IX-V (GPIb-IX-V) and integrin $\alpha_{\mathrm{IIb}}\beta_{3}$, bind to exposed endothelial matrix proteins such as von Willebrand factor (vWF) and collagen at sites of endothelial disruption [37]. In addition, tumors often present a microenvironment that mimics such vascular injury: their angiogenic vasculature is disorganized and hyperpermeable, with enlarged interendothelial gaps and an exposed basement membrane, while tumor cells and the associated stroma release potent platelet-activating signals (e.g., thrombin, adenosine diphosphate). This pathological mimicry recruits platelets and causes them to aggregate precisely at desired delivery sites [38,39]. For this reason, platelet-mimetic nanocarriers have gained attention because they retain this targeting ability [17,40].

Their small size gives them a mechanical edge, allowing passage through tight barriers like the BBB and the blood–retinal barrier. This opens up new routes to treat brain tumors or retinoblastoma [41]. Platelets circulate for 5–7 days, allowing sustained drug release after loading [23]. Inside, they carry granules (α-granules, dense granules) that can be loaded with drugs for controlled release [42], and their membrane is rich in receptors that are suitable for targeting modifications [17].

### 2.4. Blood Cell-Derived Membrane Vesicles: Biomimetic Nanocarriers

Beyond the use of whole cells, scientists have also tapped into the core functional components of blood cells by creating vesicles from their membranes [24,43]. These nano-sized bio-mimics retain important surface markers from their parent cells, but are often easier to work with and modify [44]. For example, extracellular vesicles derived from RBCs lack nuclear material, reducing genotoxicity risk and making them promising for clinical use [44]. Their smaller size (20–200 nm) helps them exploit the enhanced permeability and retention (EPR) effect for passive tumor targeting. They are also simpler to handle compared to live cells [45,46]. For instance, RBC membrane-coated nanoparticles inherit CD47’s ‘self’ signal, which extends their circulation time [47,48]. Macrophage membrane-based carriers can target metastatic sites [30], while neutrophil-derived exosomes show potential in inflammation targeting and BBB crossing [49,50]. Platelet membrane vesicles retain tumor-targeting and aggregation abilities, showing promise in pancreatic and breast cancer models [51,52].

## 3. Engineering and Loading Strategies

The key challenge is to load drugs efficiently while controlling their release and, crucially, preserving cellular function. Strategies vary by cell type but can be broadly categorized into two approaches: ex vivo modification (where cells are extracted, loaded, and reinfused) and in vivo loading (where carriers are designed to attach to circulating cells). The former allows more control; the latter is simpler and might be easier to adapt clinically.

### 3.1. RBC-Based DDSs: Encapsulation, Surface Engineering, and Controlled Release

For RBCs, drugs can be loaded inside the cell or attached to the membrane surface. Intracellular encapsulation protects drugs during circulation [20,53,54]. The osmotic gradient method is a classic and clinically established approach [55]. Briefly, RBCs are shrunk in a hypertonic solution to open temporary pores in their membrane. When they are returned to an isotonic drug-containing medium, they swell and take up the drug before the pores reseal [17]. This works well for small molecules (like paclitaxel, doxorubicin), antibiotics, and enzymes [20,56]. Electroporation is another method; it uses short, high-voltage pulses to create temporary nanopores for drug entry [57]. It is fast and suitable for large molecules like nucleic acids and proteins, but it carries a risk of cell rupture and is mainly used in research settings [17,58].

**Figure 2:**
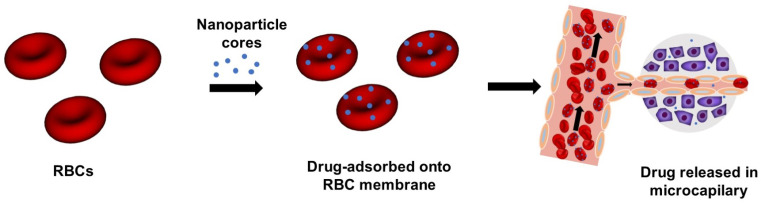
RBC-hitchhiking strategy for tissue-targeted drug delivery. Ex vivo adsorption of therapeutic nanoparticles (blue) onto the membranes of RBCs (red), followed by intravascular administration. Under shear stress in narrow capillaries, nanoparticles detach and accumulate in target tissues (e.g., tumors, purple), allowing localized drug release. The arrows indicate the workflow direction, from nanoparticle loading onto RBCs to intravascular transport and subsequent accumulation in target tissues.

Surface modification involves attaching drugs or nanocarriers directly to the RBC membrane via chemical or affinity bonds. This is useful for bulky cargo like antibodies or gene vectors [59,60,61]. Another approach is RBC-hitchhiking, where nanocarriers adsorb onto RBC surfaces. This can efficiently deliver nanoparticles to organs like the lungs while avoiding rapid clearance by the liver and spleen [47]. It is an effective way to greatly enhance nanoparticle delivery to specific tissues. The hitchhiking strategy can even be designed to release its payload under shear stress [62,63,64]. This shear-responsive release mechanism is illustrated in Figure 2. The process begins with ex vivo adsorption of drug-loaded nanoparticles onto RBC membranes. These nanoparticle-functionalized RBCs are then introduced into the bloodstream. As the RBC-carrier complexes circulate and traverse narrow capillaries, they deform under fluid shear stress and vascular wall confinement. This mechanical interaction promotes nanoparticle detachment from the RBC surface, resulting in their retention within capillary walls and subsequent accumulation in specific organs or tissues.

Recent studies have quantitatively characterized the efficiency of this detachment process. These studies show that nanoparticles remain stably adsorbed on RBC membranes under static conditions; however, upon exposure to fluid shear stress, detachment occurs in a shear-dependent manner. Specifically, at a low shear stress of approximately 1 Pa, the detachment rate ranges from 40% to 55%. When the shear stress is increased to 6 Pa (a level comparable to that encountered in pulmonary capillaries), the detachment rate increases to approximately 76% [63]. This shear-responsive detachment mechanism provides substantial in vivo therapeutic advantages. Compared to freely circulating nanoparticles, intravenous administration of RBC-hitchhiking carriers achieves a greater than 10-fold increase in pulmonary drug accumulation while also reducing accumulation in off-target organs such as the liver and spleen [47]. Moreover, when administered via carotid artery injection, approximately 10% of the delivered dose accumulates in the brain, offering a promising strategy for treating conditions such as acute stroke [62]. Collectively, these findings validate the RBC-hitchhiking approach as an efficient platform for targeted drug delivery, which leverages the intrinsic mechanical properties of RBCs to achieve organ-specific accumulation.

Crucially, the therapeutic efficacy of loaded carriers depends not only on successful delivery but also on controlled drug release [65]. For RBC-based DDSs, this release can be passive (just slow leakage over time or upon natural aging) or active. In active release, carriers respond to tumor cues like acidic pH, high glutathione levels, or specific enzymes to release drugs on demand [17,23].

### 3.2. WBC-Based DDSs: Leveraging Homing, Phagocytosis, and Bioinspired Designs

Loading WBCs often takes advantage of their natural functions like phagocytosis, migration, and adhesion. Common strategies include endocytic loading or surface conjugation, sometimes combined with nanodrugs to create hybrid systems [66].

Endocytic loading uses the phagocytic ability of macrophages and neutrophils. Simply incubating them with nanodrugs (like liposomes, polymer nanoparticles) leads to internalization, creating Trojan horse carriers [28]. These hybrids combine the long circulation and active homing of WBCs with the high drug payload of nanoparticles, helping drugs reach hypoxic tumor regions more effectively [44,67]. For example, doxorubicin-loaded neutrophils recruited by tumor chemokines accumulate better in lung cancer lesions than free drugs, and with lower toxicity [23].

Genetic engineering is key for lymphocytes. Using lentiviral vectors, T cells or natural killer (NK) cells can be engineered to express tumor-targeting receptors such as chimeric antigen receptors (CARs) and produce therapeutic agents directly at tumor sites. Surface conjugation links drugs to WBC surfaces chemically (e.g., via biotin–avidin bonds) [68]; however, care must be taken to avoid interfering with proteins essential for adhesion and migration [69]. Drug release from these carriers is often coupled with the natural life cycle of the WBCs. At inflammatory tumor sites, activation signals can induce lysosomal fusion (releasing encapsulated drugs) or trigger apoptosis, ensuring localized payload delivery [28,30].

**Figure 3:**
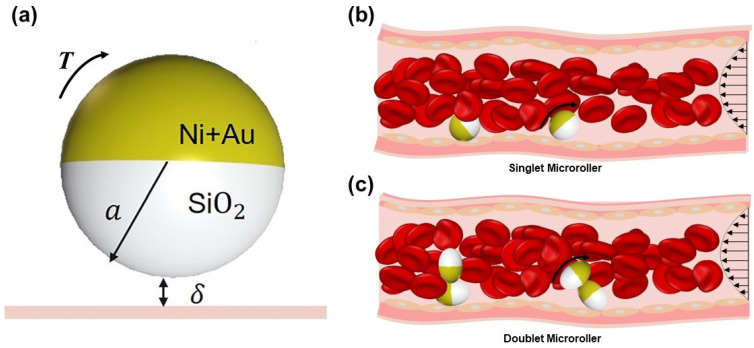
Schematic representation of WBC-inspired microrollers for active drug delivery in the bloodstream. (**a**) Schematic design of a magnetically driven Janus microroller, consisting of a silica (SiO_2_) core (white) partially coated with nickel and gold (Ni/Au, yellow) layers. The functionalized silica hemisphere carries targeting antibodies and drug payloads. The curved arrow indicates the rotational direction, and *T* indicates magnetic torque. (**b**,**c**) Illustration of singlet (**b**) and doublet (**c**) microrollers performing upstream motion with surrounding RBCs (red) along vascular walls under flow conditions. Straight arrows indicate blood flow direction; curved arrows along the vessel wall indicate the rolling direction of the microrollers.

In addition to leveraging natural WBC biology, bioengineering approaches have created synthetic micro-scale systems that mimic WBC margination and locomotion [70,71,72]. A prominent example is the magnetically driven Janus microrollers (Figure 3), which mimic the ability of WBCs to navigate along vessel walls against blood flow. These half-coated particles can be guided along vessel walls, mimicking the margination behavior of individual WBCs. Their ability to move against blood flow overcomes a key limitation of passive carriers. Functionally, they have been loaded with chemotherapeutic agent such as docetaxel, and their magnetic moiety enables photothermal heating, allowing combined chemo-thermal therapy [72]. Interestingly, their locomotion depends critically on shape: isotropic spherical rollers perform poorly on textured, vessel-like surfaces, whereas anisotropic designs (e.g., doublet chains) maintain motion efficiently [71]. Moreover, such microrollers can be monitored via MRI or photoacoustic imaging, allowing potential for real-time guidance and treatment verification. Though still in early development, these systems represent a compelling step toward actively navigable, multifunctional carriers inspired by leukocyte physiology.

### 3.3. Platelet-Based DDSs: Granule Loading, Membrane Camouflage, and Stimuli- Responsive Design

Platelets offer multiple loading routes: their intracellular granules, and their membranes for coating biomimetic nanoparticles [17]. Intracellular granule loading uses platelet storage units, namely α-granules and dense granules. Drugs can be loaded via ex vivo activation or incubation [36,42]. For example, loading a chemotherapeutic like paclitaxel into α-granules while attaching PD-1 antibodies to the membrane creates a dual-function carrier that delivers chemotherapeutic agents and blocks immune checkpoints locally [73,74].

Stimuli-responsive modification can enhance platelet functionality. Adding pH-sensitive polymers keeps platelets intact at normal blood pH but promotes degradation and drug release in the acidic tumor microenvironment [52]. Similar strategies use enzyme-sensitive linkers that are cleaved by tumor-associated proteases [73,75].

Platelet biomimetic reconstruction avoids the complexities of using live cells. Here, platelet membranes are extracted and used to coat synthetic nanoparticles. These platelet-mimetic nanoparticles retain targeting ability (they still aggregate at injury/tumor sites) but are easier to produce and load [17]. They show promise in crossing the BBB to treat glioblastoma, extending survival in animal models [41].

Natural platelet behavior establishes an ideal delivery loop: tumor vessel damage recruits platelets, and local conditions (e.g., acidity, enzymes) trigger release, allowing precise aggregation, activation, and release kinetics [41,75].

### 3.4. Blood Cell-Derived Membrane Vesicle-Based DDSs: Fabrication and Cargo Loading

The construction of biomimetic vesicles from blood cell membranes typically involves three core steps: membrane isolation, vesicle formation, and drug loading. First, cell membranes are obtained through hypotonic lysis, mechanical extrusion, or nitrogen cavitation of donor cells (e.g., RBCs, WBCs, or platelets), followed by purification via differential centrifugation to remove intracellular components [43,44,46]. These membrane fragments can then be reassembled into unilamellar vesicles by sonication, extrusion through nanoporous membranes, or microfluidic mixing. Alternatively, a more direct and widely adopted strategy is to fuse these native membranes onto pre-formed synthetic nanoparticle cores (e.g., PLGA, silica, or polymeric NPs), creating a cell membrane-camouflaged hybrid system that inherits the source cell surface markers while maintaining the structural and drug-carrying advantages of the synthetic core [7,76].

Drug loading into these vesicles can be achieved through pre-loading (incorporation during vesicle formation) or post-loading (into pre-formed vesicles). For hydrophobic drugs, passive partitioning into the lipid bilayer occurs upon simple co-incubation. Driven by the hydrophobic effect, drug molecules spontaneously migrate from the aqueous medium into the hydrophobic core of the bilayer to minimize free energy. This process requires no external energy or membrane disruption and is governed by the drug’s partition coefficient. The lipid bilayer thus acts as a reservoir for lipophilic compounds, offering advantages for membrane-acting drugs or sustained-release formulations. Hydrophilic molecules, proteins, or nucleic acids are commonly encapsulated via electroporation, which transiently disrupts the membrane to allow cargo influx, or by remote loading techniques using pH or ion gradients [46,50]. Surface conjugation via chemical linkers enables the attachment of targeting ligands (antibodies, peptides) or additional therapeutic moieties without compromising vesicle integrity [77]. This modularity in construction and loading allows the creation of tailored vesicles designed for specific diseases, combining natural homing capabilities with controlled drug release kinetics.

In short, each type of blood cell brings its own functional strengths to drug delivery. As summarized in Table 2, RBCs offer long circulation and innate biocompatibility, WBCs actively home to tumors and infiltrate deep tissues, platelets can slip through biological barriers and accumulate precisely where needed, and cell-derived membrane vesicles retain biomimetic properties while offering greater engineering flexibility. Taken together, these DDSs have been adapted to deliver a wide spectrum of therapeutic agents, positioning them as multifunctional delivery platforms for diseases ranging from cancer to cardiovascular and neurological disorders.

## 4. Therapeutic Applications in Oncology

The engineering strategies discussed above enable blood cell-based carriers to transform their inherent biological properties into therapeutic benefits. In oncology, these DDSs are promising for improving tumor-specific targeting, overcoming physiological barriers, and modulating the tumor microenvironment. The following subsections highlight the applications of these DDSs in addressing various challenges in cancer therapy.

### 4.1. RBC-Based DDSs in Tumor Therapy

RBC carriers, with their safety and long circulation, are attractive for advanced cancers [74,78]. Tumors have abnormal, highly branched microvascular networks. While this promotes tumor growth, it often hinders conventional therapies. Interestingly, RBC-based DDSs can leverage these same vascular irregularities for targeted drug transport [62,63,79]. Studies investigating the shear-induced detachment of nanoparticles from RBC surfaces under flow have shown that nanoparticles stay attached without fluid forces. But under shear stress, detachment occurs in a dose-dependent manner [63]. Using autologous RBCs loaded with immunostimulants in patients with advanced lung, colorectal, and cervical cancers resulted in a notable tumor response rate, including cases of complete remission, without severe side effects [74]. The mechanism likely involves the natural clearance of RBCs via the splenic route, where delivered drugs can activate systemic immune responses against tumors [80].

The use of RBCs is also opening up new possibilities in gene therapy [15,17]. The multiscale approach using RBC-mediated hitchhiking and vascular endothelium leakage (MARVEL) system used RBCs co-loaded with adeno-associated virus (AAV) gene vectors and vascular endothelial growth factor (VEGF) [22]. RBCs homed to the lungs, and VEGF enhanced AAV penetration into deep lung tissue, reportedly boosting delivery efficiency several-fold with minimal off-target effects [17,47].

RBCs can also help prevent tumor metastasis. Drug-loaded RBCs circulate and continuously release drugs to kill circulating tumor cells (CTCs), while surface modifications can enable CTC capture, preventing them from settlement in new organs [63]. In preclinical models of breast and prostate cancer metastasis, this strategy significantly reduced metastatic lesion burden.

### 4.2. WBC-Based DDSs in Tumor Therapy

WBC-based DDSs excel in active targeting and deep tissue penetration, which is key for treating hypoxic tumors and achieving chemo-immunotherapy synergy. The macrophage Trojan horse strategy is particularly notable for deep tumors [16]. Macrophages naturally migrate into hypoxic regions; when loaded with nanoformulated chemotherapeutic agents, they can release their payload in response to hypoxia. In preclinical models of pancreatic cancer, macrophage-mediated delivery of cisplatin markedly enhanced drug accumulation within hypoxic tumor areas and demonstrated substantial tumor suppression, whereas free cisplatin failed. They can also modulate the tumor microenvironment to synergize with other treatments [81].

Neutrophils, with their natural chemotaxis to inflammatory sites, have been used to deliver drugs to brain tumors or ischemic heart tissue, showing better targeting and outcomes in preclinical models [33]. Their ability to migrate to sites of damage and cross biological barriers has been used to deliver chemotherapeutic agents across the BBB for glioblastoma in animal studies. They are also effective against metastases, homing to lung metastases in breast cancer models [36]. Neutrophil membrane-mimetic nanoparticles show potential in inflammation-targeted therapy [49].

Lymphocyte-mediated systems, especially engineered CAR-T cells carrying drugs, aim to combine targeted killing with direct chemotherapy. This could address tumor heterogeneity and immune suppression in the microenvironment [36,81]. For example, engineering CAR-T cells to carry cytotoxic drugs (e.g., diphtheria toxin) allows drug release upon tumor antigen recognition, killing heterogeneous tumor cells that might be missed by CAR-T cells alone [23].

### 4.3. Platelet-Based DDSs in Tumor Therapy

**Figure 4:**
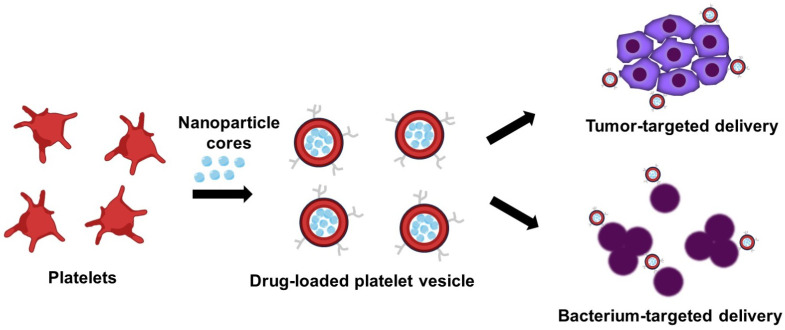
Platelet-derived nanovesicles for targeted therapeutic delivery. Preparation involves isolation of platelet membranes (red), vesicle reconstitution, and remote loading of small-molecule drugs (cyan), as guided by the arrows. The resulting nanovesicles preserve platelet membrane proteins, facilitating active targeting to disease-relevant sites such as tumors (purple) and infectious foci (deep purple).

Platelet-based DDSs use their unique abilities to accumulate at the tumor vasculature and penetrate barriers, showing promise for refractory cancers and metastatic disease [24]. Their natural tropism for vascular injury helps overcome dense stroma, as in pancreatic cancer [82,83]. This addresses the poor drug penetration into fibrotic tumor stroma, where traditional chemotherapy often fails (Figure 4) [82,84]. For example, gemcitabine-loaded platelets exploit natural tumor vascular accumulation to cross stromal barriers, enhancing local drug release at tumor sites [46].

Beyond that, platelet-based DDSs show potential in intercepting metastasis. Platelets naturally adhere to CTCs in the bloodstream; engineering them to carry anti-adhesive or cytotoxic agents offers a strategy to disrupt CTC survival and impede colonization of distant organs [20]. In addition, the active recruitment of platelets to sites of angiogenesis provides a rationale for using them to deliver antiangiogenic drugs directly to developing metastatic lesions [57]. Preclinical studies, including those targeting bone metastases, suggest promising directions for reducing metastatic burden [51,52].

Clinical studies have reported improved treatment responses and extended median survival, with a better side effect profile compared to conventional chemotherapy [45,47]. In glioblastoma, platelet delivery bypasses the BBB, which blocks most small-molecule drugs [85]. Because of their small size and response to thrombin signals from glioblastoma tissue, platelets cross the BBB and deliver drugs directly into the brain. Preclinical studies show that temozolomide-loaded platelets achieve much higher intracranial drug concentrations than the free drug, leading to extended survival without significant neurotoxicity [41]. Platelet-mimetic nanocarriers also show efficacy in treating brain metastases; for instance, platelet membrane vesicles demonstrate targeted delivery potential [51].

## 5. Emerging Applications Across Disease Settings

The therapeutic potential of blood cell-based DDSs extends well beyond oncology. By leveraging the same innate targeting, biocompatibility, and barrier-penetrating abilities, these platforms are being actively explored for a range of inflammatory, cardiovascular, and neurological disorders.

In inflammatory diseases, WBC-based DDSs enable precise drug delivery to inflammation sites [28,66]. Conditions like rheumatoid arthritis and Crohn’s disease involve inflammatory cell infiltration and cytokine release, which naturally recruit neutrophils via chemokine signaling [44]. Loading these cells with anti-inflammatory agents enables sustained local drug release, enhancing efficacy while minimizing systemic exposure [23]. In preclinical models of rheumatoid arthritis, dexamethasone-loaded neutrophils were observed to substantially reduce joint inflammation, while avoiding the systemic side effects typically associated with long-term systemic steroid use [33].

For cardiovascular diseases, both RBC- and platelet-based DDSs offer advantages [44]. In atherosclerosis, statin-loaded RBCs provide prolonged systemic drug exposure while adhering to atherosclerotic plaques, allowing localized drug enrichment that may reduce plaque rupture risk [9,23]. Platelet carriers naturally target vascular injury sites and can be loaded with antiplatelet agents or vascular repair factors to prevent thrombosis and promote endothelial healing [73,75]. Biomimetic nanoparticles derived from platelet membranes have shown progress in selectively targeting atherosclerotic lesions and supporting vascular repair [23,47,86].

In neurological disorders, platelet-based DDSs offer a promising way to overcome the BBB, a major obstacle in treating Alzheimer’s and Parkinson’s diseases [33,34]. Platelets naturally cross the BBB and, when loaded with neurotrophic factors such as BDNF, can release therapeutics directly into brain tissue to support neurons [17]. Preclinical Alzheimer’s disease models showed significant cognitive improvement following such interventions [87]. In addition, intranasally administered RBC membrane-coated gene carriers traverse the BBB, providing a non-invasive platform for neurologic gene therapy [44]. These examples highlight the versatility of blood cell-based DDSs. By leveraging inherent biological functions, researchers are opening new therapeutic avenues across diverse disease areas.

## 6. Current Challenges and Mitigation Strategies

Despite progress, real-world application faces challenges in loading efficiency, scalable manufacturing, precise targeting, and long-term safety. Solving these will require multidisciplinary effort.

### 6.1. Key Challenges

Balancing Drug Loading and Cell Viability: Different loading strategies have inherent limitations. The osmotic gradient method for RBCs shows low efficiency for large molecules; electroporation carries a risk of irreversible membrane damage and hemolysis [55,88]. WBC endocytic loading depends on cell viability, and apoptosis during ex vivo incubation can cause efficiency fluctuations [89]. Platelet granule capacity is limited; excessive drug loading may impair activation responses and reduce targeting ability [42]. Platelets’ short circulation (5–7 days) limits drug duration, and modifications may shorten it further [41]. Drug loading can damage membrane structure and mechanical properties, affecting circulation and targeting [52]. Balancing high loading efficiency with cell viability remains a core technical hurdle [90].

Scalable Production and Clinical Compatibility: Most current technologies rely on autologous cell ex vivo modification. This requires individualized preparation per patient, which is time-consuming, costly, and complex, making it not suited for large-scale use [91]. Allogeneic blood cells offer scale but risk immune rejection; even with membrane modification, immune reactions may occur. Maintaining sterility and cell viability during ex vivo processing is challenging in basic medical settings, limiting access [80].

Targeting Specificity and Off-Target Effects: Some DDSs rely on natural traits, which may lack precision [92]. RBC spleen homing could lead to excessive drug accumulation in the spleen, causing functional damage [74]. WBCs may accumulate at normal inflammation sites besides tumors, leading to off-target effects [93]. Platelets may undergo nonspecific aggregation in thrombosis patients, raising thrombotic risk [36]. Tumor heterogeneity means some tumors may not recruit blood cells effectively, reducing delivery efficiency [80].

Long-Term Safety and Biocompatibility Risks: Though autologous blood cell carriers are relatively safe, long-term infusion of modified cells may affect normal hematopoiesis and circulatory homeostasis [17]. For example, repeated administration of drug-loaded RBCs may affect overall RBC homeostasis, with potential implications for oxygen delivery [15]. Modified WBCs may become abnormally activated, triggering autoimmune responses [94]. Drug-loaded platelets could impair normal hemostasis, increasing bleeding risk [56]. Current studies have short follow-up periods, so a lack of long-term safety data is a major barrier to clinical translation.

Insufficient Control over Drug Release Kinetics: Most DDSs rely on passive lysis or simple microenvironment responses, making it hard to control release rate and dose precisely [95]. This could cause excessive drug concentration at tumor sites or insufficient concentration, leading to toxicity or reduced efficacy, respectively [54]. Some carriers leak drugs prematurely during circulation, reducing targeting efficiency and increasing systemic toxicity [27].

### 6.2. Strategies to Overcome Translational Barriers

Addressing the multifaceted challenges outlined above requires integrated innovations across material science, mechanical engineering, biomedical engineering, and clinical medicine. Encouragingly, recent clinical advances demonstrate that these challenges can be overcome. A Phase 1 trial developed αPD1-Ery, an erythrocyte-anti-PD1 antibody conjugate, for treating advanced solid tumors resistant to checkpoint inhibitors [96]. This study demonstrated that autologous RBCs could be engineered ex vivo to stably display therapeutic antibodies while preserving critical cellular properties (including deformability) and CD47-mediated immune evasion. Upon reinfusion, these carriers achieved prolonged circulation and an impressive 78.6% disease control rate with a favorable safety profile in patients with prior immunotherapy resistance. This trial validates key principles discussed in this review—maintaining native cell mechanics, feasibility of GMP-compliant manufacturing, and clinical therapeutic potential—serving as both a compelling proof-of-concept and an inspiration for the field. Building on this progress, the following strategies highlight promising directions to overcome the current limitations in loading efficiency, scalable production, targeting precision, long-term safety, and controlled drug release.

Advancing High-Efficiency Loading Techniques: To address the challenge of balancing high drug loading with cell viability and function, gentle and cell-friendly loading methods are being developed [97,98,99]. These approaches aim to maximize payload capacity while preserving essential cellular functions. For example, ultrasound-assisted osmotic gradient methods use low-intensity ultrasound to reversibly enhance RBC membrane permeability, achieving high loading efficiency for large molecules without compromising cellular deformability [100,101]. For WBCs, microfluidic chip-mediated endocytic loading enables precise control over incubation conditions, stabilizing loading efficiency while minimizing apoptosis [97]. In platelets, genetic or bio-orthogonal engineering strategies offer the potential to expand granule storage capacity for high-dose drugs without disrupting native functionality [102]. Furthermore, co-loading approaches, such as functionalizing the RBC surface with targeting ligands while loading chemotherapeutic agents inside, can combine precise targeting with enhanced therapeutic potency [81,99].

Enabling Scalable Manufacturing: To overcome the scalability and cost limitations associated with individualized, ex vivo modification, emerging production platforms are being developed to transition from patient-specific batch processing to more standardized, scalable workflows [103,104]. For instance, a recently reported “soft extrusion” method enables highly reproducible, bench-scale production of RBC-derived extracellular vesicle mimetics with demonstrated potential for clinical scale-up [104]. Parallel advances in bioreactor-based expansion of erythroid cells for transfusion purposes demonstrate the feasibility of large-scale, GMP-compliant production of RBC-derived products, offering technological models for the manufacture of blood cell-based drug carriers [105,106]. Perhaps most radically, in vivo loading strategies, in which systemically administered nanocarriers bind directly to circulating blood cells, could bypass ex vivo manipulation entirely. This concept has been validated in multiple preclinical models using RBC-hitchhiking and related approaches, demonstrating greatly enhanced feasibility for broad clinical access [47,62,63].

Engineering Precision Targeting with Multiplexed Strategies: Improving specificity while mitigating off-target effects requires building upon natural homing mechanisms through sophisticated surface engineering [107,108,109]. For example, RBCs modified with tumor-specific antibodies combine their inherent splenic tropism with active tumor recognition capabilities. Similarly, engineering WBCs to express receptors for tumor-specific cues can enhance their recruitment to hypoxic niches within tumors. Platelets decorated with bone-homing peptides enable precise delivery to metastatic bone lesions [110]. To address tumor heterogeneity, carriers can be engineered with antibodies targeting multiple tumor antigens, thereby broadening targeting coverage [61]. It should be noted, however, that experimental demonstration of multiplexed antibody display on live blood cell carriers remains limited; current evidence is primarily derived from biomimetic membrane-coated nanoparticles or bispecific antibody designs that suggest this strategy in living cellular systems [111,112].

Ensuring Long-term Safety and Biocompatibility: Proactive management of long-term biocompatibility risks requires rigorous preclinical assessment and rational carrier design. Extended clinical follow-up studies are essential to evaluate the impact of engineered cells on hematopoiesis, circulatory dynamics, and immune homeostasis; however, such data remain scarce for blood cell carriers. A 100-day tracking study of polymer-grafted RBCs showed no immunogenicity at low graft concentrations, but elevated iron deposits and CD45+ cell accumulation in the spleen were observed at high concentrations, highlighting the need for dose optimization and longitudinal surveillance [113]. To mitigate bleeding or thrombosis risks, built-in regulatory circuits can be incorporated. For platelet-based DDSs, co-delivery of low-dose anticoagulants has been shown to achieve anti-thrombotic efficacy with low hemorrhagic risk, balancing targeted aggregation and systemic hemostasis [114,115,116].

Designing Intelligent and Multi-Responsive Release Systems: To address the limitations of passive or simple responsive release, next-generation blood cell carriers are being tailored to respond to multiple tumor microenvironmental signals (e.g., acidic pH, hypoxia, elevated glutathione levels). Such multi-responsive systems can restrict drug release almost exclusively to tumor sites, minimizing off-target leakage [117]. Externally triggered systems employing magnetic or light-responsive components offer precise, on-demand control over release timing and dosage, supporting personalized therapy strategies [47,70,118]. Additionally, tuning release kinetics through advanced membrane modifications or drug encapsulation materials can help sustain effective intratumoral drug levels [85,119]. The development of intelligent release systems responsive to external stimuli (e.g., magnetism, light, ultrasound) represents a cutting-edge direction for achieving spatiotemporal control over drug delivery [120,121,122].

## 7. Conclusions and Future Perspectives

Blood cell-based drug delivery systems (DDSs) represent a rapidly evolving frontier that brings together cell biology, nanotechnology, and biomechanics to address key limitations of conventional drug carriers. As reviewed, red blood cells (RBCs), white blood cells (WBCs), platelets, and their membrane-derived vesicles each possess distinct functional properties. These features enable researchers to design more tailored and effective delivery strategies.

Significant progress has been achieved, especially in early-stage studies and animal models. Nevertheless, the translation of these systems into clinical practice still faces challenges. These include optimizing drug loading without harming the cells, establishing scalable and reproducible manufacturing processes, enhancing targeting specificity, and thoroughly evaluating long-term safety profiles.

Moving forward, the translation of these promising systems into clinical practice will require focused efforts to overcome the identified challenges related to manufacturing, safety, and spatiotemporal control. The future focus will likely shift toward integrated, more intelligent designs: targeting using multiple signals, release mechanisms that respond to the body’s own cues, and platforms that combine drugs with immunotherapy or gene therapy. Addressing these challenges will require ongoing collaboration across materials science, mechanical engineering, biomedical engineering, and clinical medicine.

In summary, blood cell-based DDSs offer a promising and biologically informed strategy for targeted treatments. With continued refinement and careful clinical validation, these systems could offer meaningful advances not only in cancer, but also in inflammatory conditions and neurological disorders.

## Figures and Tables

**Table 1:** Comparative analysis of carrier systems for drug delivery. Key parameters include biocompatibility, stability, drug-loading capacity, and circulation duration. Blood cell-based carriers exhibit superior biocompatibility and extended circulation, although their drug-loading capacity is moderate due to cell volume limitations.

Carrier Type	Biocompatibility	Stability	Drug-Loading Capacity	Circulation Duration
Nanoparticle-based DDS	Variable (material-dependent)	High (but degradation-sensitive)	High	Short (rapid RES clearance)
Liposome-based DDS	High (biomimetic membrane)	Moderate (oxidation-sensitive)	Moderate to High	Moderate (improved by PEGylation)
Blood Cell-based DDS	**High** (autologous, ‘self’ markers)	**High** (but sensitive to handling)	Moderate (cell volume limited)	**Long** (comparable to natural cell lifespan)

**Table 2:** Summary of blood cell-based drug delivery strategies. A comparative overview of RBCs, WBCs, platelets, and blood cell-derived membrane vesicles, highlighting their core advantages, key challenges, and potential clinical uses.

Carrier Type	Core Advantages	Key Challenges	Main Applications
RBCs	Long circulation time, high biocompatibility, large loading capacity	Limited targeting capability, modifications may affect lifespan	Chronic diseases, enzyme replacement therapy, palliative care for solid tumors
WBCs	Active inflammation/tumor homing, deep tissue penetration	Complex isolation and modification, potential immunosuppression	Autoimmune diseases, infections, solid tumors (esp. hypoxic regions)
Platelets	Natural injury/thrombus targeting, ability to cross biological barriers	Relatively short lifespan, demanding storage requirements	Thrombotic diseases, tumors (esp. pancreatic cancer, glioblastoma)
Blood Cell-derived Vesicles	Low immunogenicity, retains source cell targeting, easy to modify	Heterogeneity, difficulties in isolation and purification	Neurological disorders, cancer, inflammatory diseases

## Data Availability

Data sharing is not applicable to this article as no new data were created or analyzed in this study.

## Abbreviations

The following abbreviations are used in this manuscript:

AAV	Adeno-associated virus
ADP	Adenosine diphosphate
BBB	Blood–brain barrier
BDNF	Brain-derived neurotrophic factor
CARs	Chimeric antigen receptors
CTCs	Circulating tumor cells
CXCL12	C-X-C motif chemokine ligand 12
DDS	Drug delivery system
EGFR	Epidermal growth factor receptor
EPR	Enhanced permeability and retention
HER2	Human epidermal growth factor receptor 2
IL-6	Interleukin-6
MARVEL	Multiscale approach using RBC-mediated hitchhiking and vascular endothelium leakage
NK	Natural killer
PD-1	Programmed cell death protein 1
RBCs	Red blood cells
TNF-α	Tumor necrosis factor alpha
VEGF	Vascular endothelial growth factor
vWF	von Willebrand factor
WBCs	White blood cells
